# Design of Real-Time Extremum-Seeking Controller-Based Modelling for Optimizing *MRR* in Low Power EDM

**DOI:** 10.3390/ma16010434

**Published:** 2023-01-03

**Authors:** Mohamed Rabik Mohamed Ismail, Muthuramalingam Thangaraj, Panagiotis Karmiris-Obratański, Emmanouil Papazoglou, Nikolaos Karkalos

**Affiliations:** 1 Department of Mechatronics Engineering, SRM Institute of Science and Technology, SRM Nagar, Chennai 603203, TN, India; 2 Department of Manufacturing Systems, Faculty of Mechanical Engineering and Robotics, AGH University of Science and Technology, 30-059 Cracow, Poland; 3 School of Mechanical Engineering—Laboratory of Manufacturing Technology, National Technical University of Athens, 10682 Athens, Greece

**Keywords:** EDM, ESC, optimal, *MRR*, discharge

## Abstract

Electric discharge machining (EDM) is one of the non-conventional machining processes that supports machining for high-strength and wear-resistant materials. It is a challenging task to select the process parameters in real-time to maximize the material removal rate since real-time process trials are expensive and the EDM process is stochastic. For the ease of finding process parameters, a modelling of the EDM process is proposed. Due to the non-linear relationship between the material removal rate (*MRR*) and discharge time, a model-free adaptive extremum-seeking controller (ESC) is proposed in the feedback path of the EDM process for finding an optimal value of the discharge time at which the maximum material removal rate can be achieved. The results of the model show a performance that is closer to the actual process by choosing steel workpieces and copper electrodes. The proposed model offers a lower error rate when compared with actual experimental process data. When compared to manual searching for an optimal point, extreme seeking online searching performed better as per the experimental results. It was observed that the experimental validation also proved that the ESC can produce a large *MRR* by tracking the extremum control. The present study has been limited to only the *MRR*, but it is also possible to implement such algorithms for more than one response parameter optimization in future studies. In such cases the performance measures of the process could be further enhanced, which could be used for a real-time complex die- and mold-making process using EDM.

## 1. Introduction

A small gap between an electrode and a workpiece immersed in dielectric fluid forms a structure for electrical discharge machining (EDM), a method of removing metal by creating controlled sparks. EDM can be used for several hole-fabrication methods due to its non-contact nature [1,2]. Since the discharge energy determines the material removal mechanism in the process, the gap voltage and discharge current are considered as crucial factors while analyzing the process mechanism. During experimental trials, the lack of stability in an EDM gap profile has been found to be a critical problem. For example, numerous studies on the EDM waveform have been conducted [3,4,5], but none of them were able to find the ideal profile due to a highly stochastic and complicated EDM mechanism which could result in unwanted drawbacks including adhesion, short-circuiting, and cavitation [6,7]. In accordance with the terms of the invention, the described models for the EDM process for creating a mechanism for removing material were based on energy and heat transfer equations and were employed for the input voltage, peak current, and pulse-on-time of the machining setup as process parameters [8,9]. There have been numerous reported attempts to simulate the process’s machining rate using analytical, geometrical, statistical, or empirical methods [8,9,10]. There have also been a number of different methods for predicting the *MRR* using a combination of optimization and statistical techniques, including the integration of an artificial neural network (ANN) with a particle swarm algorithm [11], and ANN-based models for *MRR* prediction and the use of an artificial bee colony algorithm to optimize the parametric combinations for a better *MRR* with less surface roughness [12,13,14]. According to the theoretical improvements that have been published, improving the accuracy in estimating the actual discharge power used to generate heat at a workpiece surface during a spark’s occurrence is essential to accurately anticipate the rate of material removal (*MRR*) in EDM [15,16]. By evaluating the real-time V-I waveforms obtained during an EDM operation to calculate the discharge power, the model integrates the actual discharge conditions. Meanwhile, the discharge power density is established by the model to depend on the applied heat flux and discharge gap [17]. In [18], the surface roughness and material removal rate (*MRR*) were set as the output responses for the machining. Taguchi’s robust design plans the machining process, and the Taguchi method can be used to obtain the best *MRR*. A vibration applied to a workpiece during EDM considerably improves the material removal rate (*MRR*) and surface roughness (SR) [19]. Moreover, a modelling of the whole EDM process and modelling with dynamic conditions were performed and the output parameter *MRR* was calculated and compared with existing results for justifying the models [20,21,22]. To produce the electrodes for die-sinking EDM, a FDM (fused deposition modelling) was used, and its significance and applications are highlighted in this study (i.e., electrical discharge machining). A literature-based survey was also carried out, and it was found that there has not been much discussion of FDM’s suitability for producing EDM electrodes [23].

Some of the literature has been examined to show that *MRR* is a non-linear function of the discharge time. Some mathematical models of the material removal rate process have been developed in the past based on the boundary condition of the plasma formed between a cathode (i.e., a workpiece) and an anode (i.e., an electrode). These two models, one for cathode erosion and the other for anode erosion, were based on the thermophysical properties of plasma at temperatures ranging from a solid to a liquid melt [24,25]. The complicated link between a material and the plasma was, nevertheless, shown in these models. In [26,27], a mathematical model used a dimensional analysis to find the crucial electrical and physical factors that affect the rate at which material is removed. The *MRR* was shown in these studies as a non-linear function of the discharge time. Due to the recent proof of its first rigorous local stability analysis for an ES scheme, extremum-seeking control—a model-free and adaptive optimization technique—has gained more interest [28]. This method can be used successfully for non-linear effects such as battery equalization under different external conditions, maximum power seeking in photo-voltaics, and as an anti-lock braking system under dynamic conditions [29]. The extremum-seeking control and its procedural methods have been explained in [30,31].

From a detailed literature review, it was evident that little attention had been provided on adopting different control approaches in unconventional machining processes, and there was no work available on adopting an extremum-seeking control (ESC) for the maximum removal rate by searching an online optimal discharge time under dynamic conditions. Hence, the present investigation was made. In the present study, an attempt was made to implement an ESC-based control for maximizing the *MRR* in the EDM process.

## 2. Experimental Methodology

**Figure 1:**
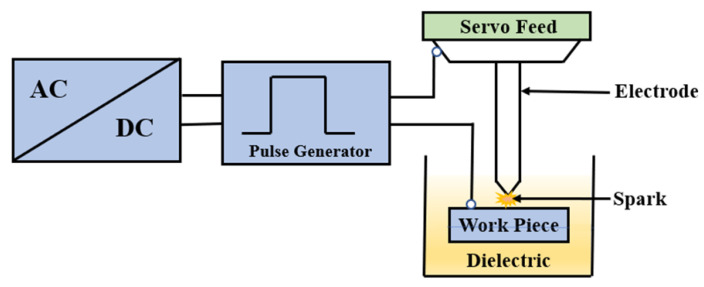
Functional block diagram of the proposed system.

**Figure 2:**
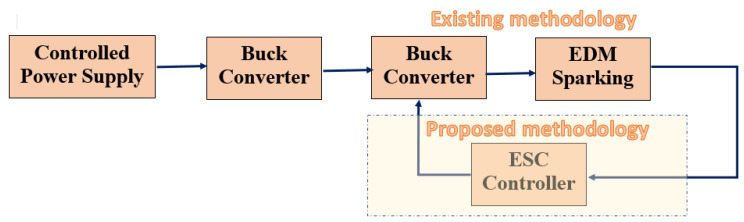
Proposed low-power EDM with ESC controller in feedback path.

The functional block diagram is represented in Figure 1. In the EDM process, the tool electrode and specimen were separated by an air gap and immersed in a dielectric medium, while controlled pulses, with the help of a converter and pulse generators, were supplied across the machining zone. Discharge sparks were formulated in the machining zone due to a dielectric breakdown in the EDM arrangement as shown in Figure 1. The tool electrode could be moved up and down using a servo tool feed control. The tool was connected with a negative polarity whereas the specimen was connected with a positive polarity [32].The blocks of the conventional EDM process with an additional buck converter and ESC in the feedback path for a low-power conversion are shown in Figure 2. The determined *MRR* was provided to the EDM process through an ESC controller. The ESC finds an optimal input for the EDM process for maximizing the *MRR*. In the existing methodology, the controlled spark pulses from the power supply was provided to a buck converter for signal conditioning to produce sparking in the EDM process. Since the EDM process is a stochastic machining process, there is always a non-linearity of the *MRR* possible with the discharge time and it was necessary to introduce a new control methodology to eliminate the non-linearity as much as possible. Hence, this was needed for formulating the extremum-seeking control in the process with a perturbation signal for optimal searching. The extremum-seeking control could be applied to the EDM process with the appropriate controller parameters.

### 2.1. Modelling and Design of a Low-Power EDM Process

**Figure 3:**
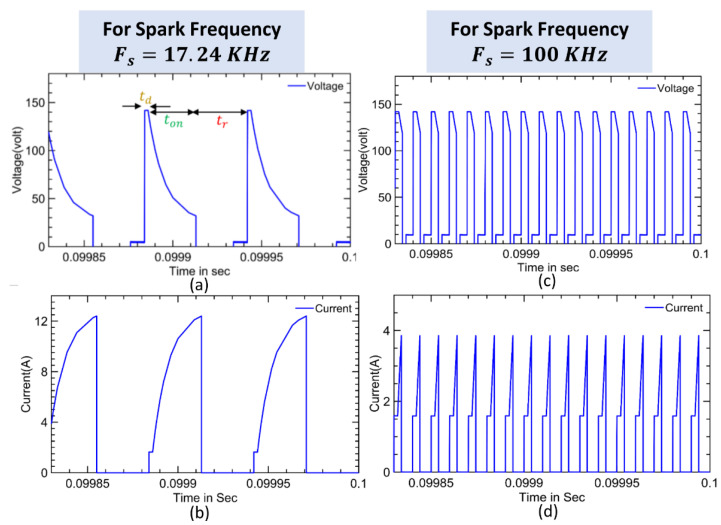
Gap voltage and current waveforms during the EDM process (**a**,**b**). For the spark frequency (F_s_) = 17.24 KHz, and (**c**,**d**) for the spark frequency (F_s_) = 100 KHz.

The modelling of the low-power EDM process can be represented with three important subblocks, namely, a power supply design, buck converter design, and EDM process modelling with *R* and *L* circuits. The pulses for the EDM process can be generated comprising a unit of power supply and a buck converter. It mainly consists of an AC source, a bridge rectifier, a capacitor as the filter, and a pulsed (P_1_) MOSFET transistor as a switching circuit to generate pulses, as shown in Figure 3. The importance of switched and low-power converters is explained in [33]. In this model, a low-power conversion was achieved by a proper design of the buck converters pulsed with P_2_ with a continuous conduction mode of conversion. In the design, the inductor (*L_b_*) and capacitor (*C*_2_) can be selected based on the maximum load conditions of the EDM process and the values are given by:

(1) $$R_{L}=\frac{2L_{b}}{\left(1-D\right)T}$$

(2) $$L_{b}=\left(\frac{\left(V_{s}-V_{i}\right)}{\Delta I_{L}}\right)D\cdot T$$

(3) $$C_{2}\geq C_{min}=\frac{1-D}{8\cdot L_{b}\left(\frac{\Delta V_{i}}{V_{i}}\right)f^{2}}$$

where *T* = 1/*f*, *f* is the frequency of the pulse P_2_; *D* is the duty cycle of the pulse waveform P_2_; $\Delta I_{L}$ is assumed to be 30% of the maximum load current $I_{L}$ and $\Delta V_{i}$ is the ripple voltage and is assumed to be 200 mV.

### 2.2. Ignition Phase

**Figure 4:**
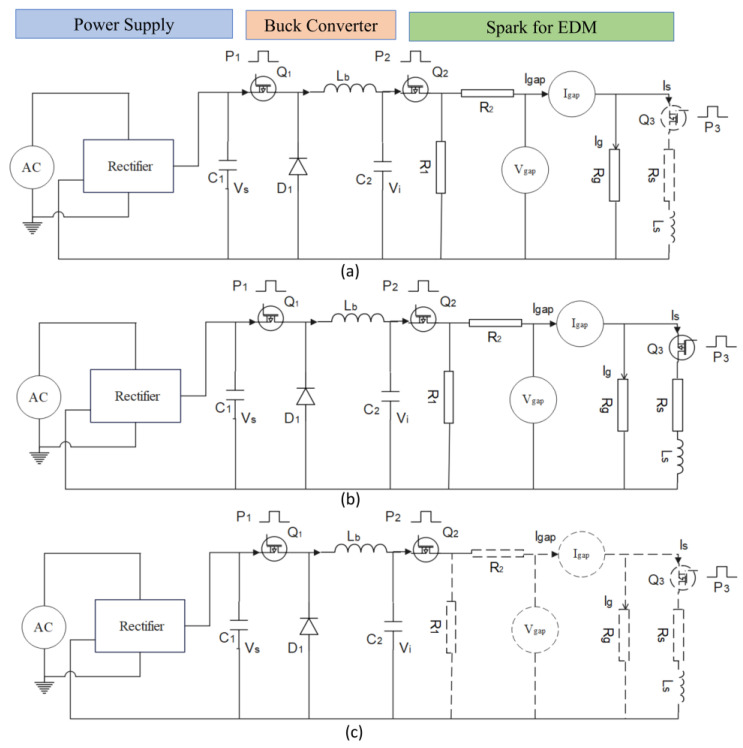
The model circuit and current path (solid lines) in the (**a**) ignition phase, (**b**) discharge phase and (**c**) recovery phase.

As previously stated, the modelling of the low-power EDM process can be represented with three important sub blocks, namely, a power supply design, buck converter design, and EDM process modelling with *R* and *L* circuits. The gap voltage and current waveforms during the EDM process for low and high spark frequencies(17.24 KHz and 100 KHz) are shown in Figure 3a–d. As illustrated in a, Figure 3a the ignition phase is carried out in the time $t_{d}$ called the delay time. A larger gap voltage creates a higher electric field between the workpiece and the electrode. A smaller delay time gives the space for a larger spark time to reach higher energy into the workpiece [8,9,17]. The switch *Q*_2_ is on and the switch *Q*_3_ is off and gives the current path as indicated by solid lines and no current flow indicated by dotted lines, as shown in Figure 4a. As the *Q*_3_ is off, *I_g_* = *V_gap_* and using Ohm’s law, *I_g_* = *V_gap_*/*R_g_* from the circuit diagram:

(4) $$\frac{\left(V_{i}-V_{gap}\right)}{R_{2}}=I_{gap}=I_{g}$$

From the above equation, it can be seen that a smaller difference of the *V_i_* and *V_gap_* leads an ideal model, and when the gap voltage is equal to the input voltage *V_gap_* ≈ *V_i_*, then the *i_gap_* ≈ 0.

### 2.3. Discharge Phase

The discharge phase of the EDM process occurs during the interval $t_{on}$ as shown in Figure 3a. The nature of the isolation of the dielectric is broken down, and the current increases rapidly while the voltage is falling. Then, a spark is formed due to a larger gap current, *I_g_*, with the lowest gap voltage being $V_{on}$. The MOSFET transistors $Q_{2}$ and $Q_{3}$ are switched to an on condition and the current flow of the components is indicated as the solid lines in Figure 4b. A series of connections of $L_{s}$ and $R_{s}$ are connected in parallel with a resistor, *R_g_*. The gap voltage and current can be written from the circuit as follows.

(5) $$I_{gap}=I_{R_{s}}+I_{R_{g}}$$

(6) $$V_{gap}=I_{R_{s}}R_{s}+L_{s}\frac{dI_{R_{s}}}{dt}$$

Taking a Laplace transform for (6) and solving it for the current $I_{R_{s}}:$

$$V_{gap}\left(s\right)=I_{R_{s}}\left(s\right)\cdot R_{s}+L_{s}s\cdot I_{R_{s}}\left(s\right)\;I_{R_{s}}\left(s\right)=V_{gap}\left(s\right)/\left(R_{s}+L_{s}s\right)$$

where *s* is the Laplace variable, while taking an inverse Laplace transform to find the current $I_{R_{s}}$ in the time domain:

(7) $$I_{R_{s}}=\frac{V_{gap}}{R_{s}}\left(1-e^{-\frac{R_{s}t_{d}}{L_{s}}}\right)$$

From the circuit diagram $I_{R_{g}}=\frac{V_{gap}}{R_{g}}$, then the total gap current from Equation (5) is substituted by Equation (7):

(8) $$I_{gap}=V_{gap}\left(\frac{1}{R_{s}}\left(1-e^{-\frac{R_{s}t_{d}}{L_{s}}}\right)+\frac{1}{R_{g}}\right)$$

Then, Kirchhoff’s law is applied to the loop, $V_{i},\;R_{2},\;V_{gap}$ from the circuit diagram (Figure 4b).

(9) $$V_{i}=R_{2}I_{gap}+V_{gap}$$

Substituting the Equation (8) *I_gap_* in the above equation and solving for the *V_gap_*:

(10) $$V_{gap}=\frac{R_{g}R_{on}}{R_{g}R_{2}\left(1-e^{-\frac{R_{s}t_{d}}{L_{s}}}\right)+R_{2}R_{s}+R_{g}R_{s}}V_{i}$$

The above equation explains how the input voltage is decreased during a spark and how it depends on different circuit elements.

### 2.4. Recovery Phase

This phase is occupied by the interval $t_{r}$ as shown in Figure 3a. The gap current flow is stopped, and the desirable properties of the dielectric are improved again for the next cycle. Figure 4c shows the model of the recovery phase where no current flow is indicated in the dashed line components and both the MOSFET transistors, $Q_{2}$ and $Q_{3},$ are switched off. The approximate formula to determine the energy of the pulse is given by [27].

(11) $$E=V_{gap}I_{gap}t_{on}$$

## 3. Implementation of ESC on Reducing the Non-Linearity of *MRR*

### 3.1. A Non-Linear Model of the Material Removal Rate

The material removal rate can be calculated using a dimensional analysis [26,27], and this is given by:

(12) $$MRR=C\alpha V_{on}I_{g}t_{on}F_{s}$$

The equation is used to predict the erosion rate with the help of process parameters. Where α is the material property constant and it is given by $\alpha =2\times 10^{-12}m^{3}J^{-1}$ [27]. From Equation (12), the MRR is directly proportional to the discharge time $t_{on}$ and spark frequency $F_{s}$. The dimensionless constant C, which can be calculated from the experimental data, is given by the following equation [26,27]:

(13) $$C=3.52\times 10^{-7}{\left(\frac{t_{on}}{t_{d}}\right)}^{3}-1.33\times 10^{-4}{\left(\frac{t_{on}}{t_{d}}\right)}^{2}+1.25\times 10^{-2}\left(\frac{t_{on}}{t_{d}}\right)+1.53$$

**Figure 5:**
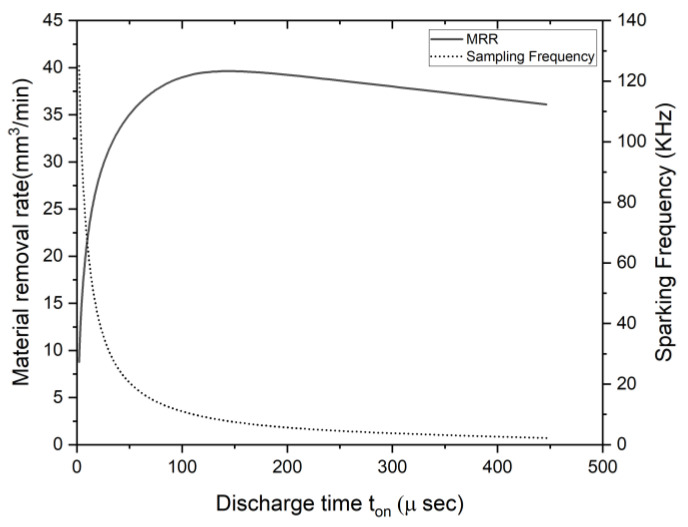
Non-linear *MRR* and spark frequency against the discharge time.

The MRR  value is plotted against the discharge time $t_{on}$ by fixing all the other parameters that are constant, as shown in Figure 5. Here, it was observed that the MRR was varying with the $t_{on}$ in a non-linear manner. The *MRR* was high at one optimal value of the $t_{on}$. This paper aimed to find the unknown value of that optimal point, $t_{on},$ under dynamic conditions. Additionally, manual tracking can be used for searching the optimal point, but it is not very helpful when the conditions are changed. In this paper, an extremum-seeking method was used for finding the optimal point at which the removal rate was maximum in an online mode.

### 3.2. A Non-Linear Model of the Material Removal Rate

**Figure 6:**
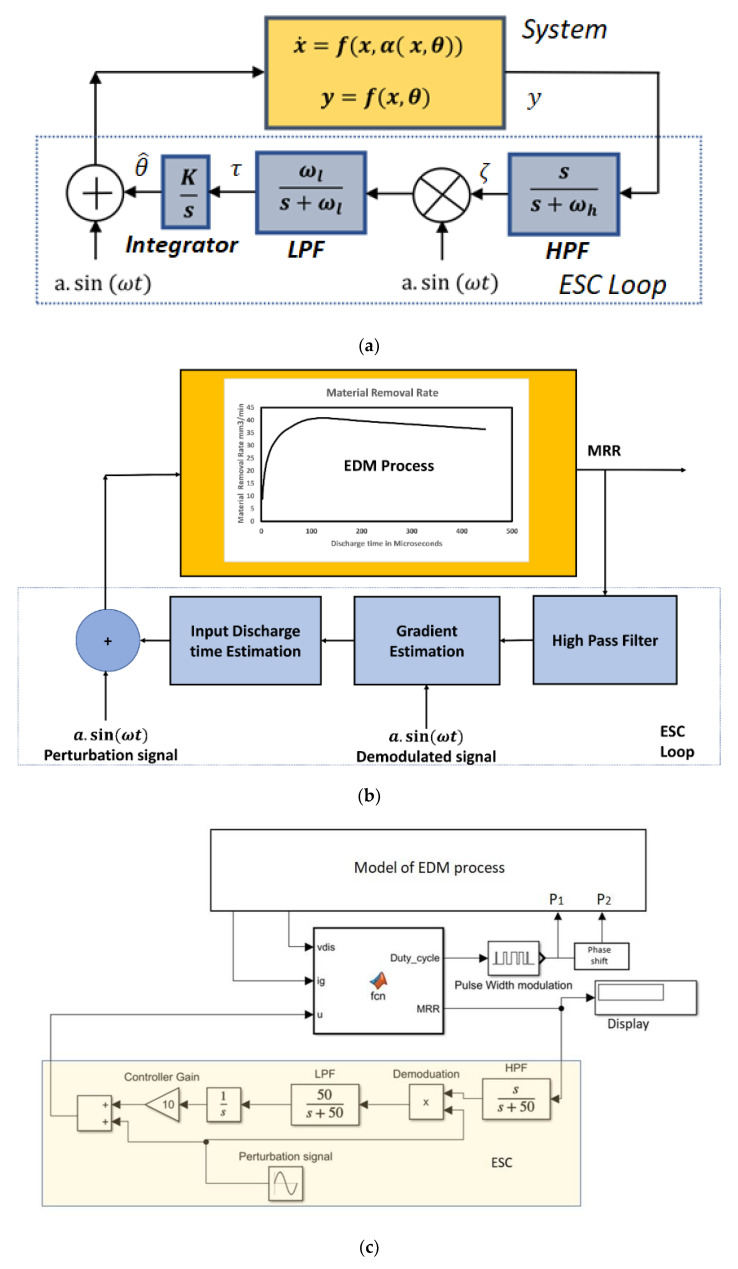
Methodology of extremum-seeking control for the EDM process. (**a**) Extremum-seeking control structure in a closed loop. (**b**) A detailed schematic of the proposed extremum-seeking control for the EDM process. (**c**) A detailed schematic of the proposed extremum-seeking control for the EDM process.

An extremum-seeking controller structure with a system is shown in Figure 6a. A detailed diagram of the proposed ESC control for the EDM process for searching for an optimal discharge time is shown in Figure 6b. The *MRR* value can be calculated from the model parameters, and fed to the ESC controller, and since the relationship between the discharge time and the material removal rate is non-linear, as given in Equations (12) and (13), this non-linear control is preferred. The calculated material removal rate from the process output is then given as the feedback input for the controller, and it is assumed to be the cost function *J*(*θ*). Additionally, the *θ* input is assigned as the discharge time $t_{on}$. Furthermore, the gradient information is extracted using HPF, demodulation, and LPF. The integrated gradient information with a controller gain was given the latest discharge period input for the EDM process. As explained previously, the control input for the EDM is perturbed with a sinusoidal signal before applying it to the EDM process, as explained previously. The model parameters of the EDM, as given in Figure 6c, and the perturbed discharge time are then provided as the inputs for a Matlab function where detailed calculations of the *MRR* and the parameters of the sparking waveform are made for controlling the EDM process with the help of pulse width modulation. The system and its nature are assumed to be non-linear and have a maximum output at some optimal input of $\theta^{*}$. As illustrated in Figure 6, the input to the system here was given as $\theta =\hat{\theta }+a\cdot \sin\omega t,$ where $\hat{\theta }$ is the estimated input by the controller with a gain K, and a and ω are the amplitude and frequency of the perturbation signal, respectively. If the a is greater, then the convergence speed to reach a local optimum value will be high, but this creates larger fluctuations at the settlement point of the maximum output and leads to a larger error around the optimal point [32,33,34]. Assuming the system is static and nonlinear, its output is expressed in terms of the cost function ‘*J*’, which is given as:

(14) $$y=f\left(x,\theta \right)=J\left(\theta \right);\;\;\;y\in \mathbb{R},\;\theta \in \mathbb{R}\;$$

where the input error value is $\bar{\theta }$ and can be found using $\bar{\theta }=\theta^{*}-\hat{\theta }$.

After substituting θ and $\bar{\theta },$ using Taylor’s series expansion after neglecting the higher order terms, the equation becomes:

(15) $$y=J\left(\theta \right)\approx J\left(\theta^{*}\right)+\frac{J^{\prime \prime }\left(\theta \right)}{2}{\left(\hat{\theta }+a\cdot \sin\left(\omega t\right)-\theta^{*}\right)}^{2}$$

where $J^{\prime \prime }\left(\theta \right)$ is the second-order derivative or the accelerating signal of the $J\left(\theta \right)$ with the equation expanded. Furthermore, higher frequency terms can be allowed by a properly designed high pass filter (HPF), and the high pass filter is designed in such a way to obtain the gradient information to be added or subtracted in the input, with its cutoff frequency fixed below the perturbation frequency ω to exactly allow the variations of the output only. Its output (ζ) is as follows:

(16) $$\zeta =\frac{J^{\prime \prime }\left(\theta^{*}\right){\hat{\theta }}^{2}}{2}+a\cdot J^{\prime \prime }\left(\theta^{*}\right)\cdot \bar{\theta }\cdot \sin\left(\omega t\right)-J^{\prime \prime }\left(\theta^{*}\right)\cdot \frac{a^{2}}{4}\cdot \cos\left(2\omega t\right)$$

In the above equation, all three terms are frequency terms, and the expression is multiplied by $a\cdot \sin\left(\omega t\right)$ for the demodulation process to extract the DC term of variations. It can be sent through a low pass filter (LPF) to remove the higher-order frequency terms. Finally, the gradient information $\left(\tau \right)$ can be obtained as follows:

(17) $$\tau =-a^{2}\cdot \frac{\bar{\theta }J^{\prime \prime }\left(\theta^{*}\right)}{2}$$

The gradient information is sent to a controller which has a gain of ‘*K*’. From the equation, the derivative can be expressed as $\dot{\bar{\theta }}=-\dot{\hat{\theta }}$ and can then be expressed with a controller gain as follows:

(18) $$\dot{\bar{\theta }}=-\dot{\hat{\theta }}=-K\tau =Ka^{2}\cdot \frac{\bar{\theta }J^{\prime \prime }\left(\theta^{*}\right)}{2}$$

Solving the above equation to obtain the input error $\bar{\theta }$ around the optimal point, it can then finally be expressed as:

(19) $$\bar{\theta }\approx e^{Ka^{2}\cdot \frac{\bar{\theta }J^{\prime \prime }\left(\theta^{*}\right)\left(t-t_{0}\right)}{2}+ln{\bar{\theta }}_{0}}$$

From the above equation, the input error around the optimal point of searching depends on factors such as the controller gain K, the amplitude of the perturbation signal, and the initial error ${\bar{\theta }}_{0}$ at the time $t_{0}$. When the K is positive and the $J^{\prime \prime }\left(\theta^{*}\right)$ accelerating signal is negative while searching for the maximum output and reaching the optimal input, from the equation it can be found that the input error is zero as the time approaches infinity in Equation (19) as follows:

(20) $$\underset{t\to \infty }{\lim}\bar{\theta }=0$$

## 4. Results and Discussion

### 4.1. Experimental Simulation

**Figure 7:**
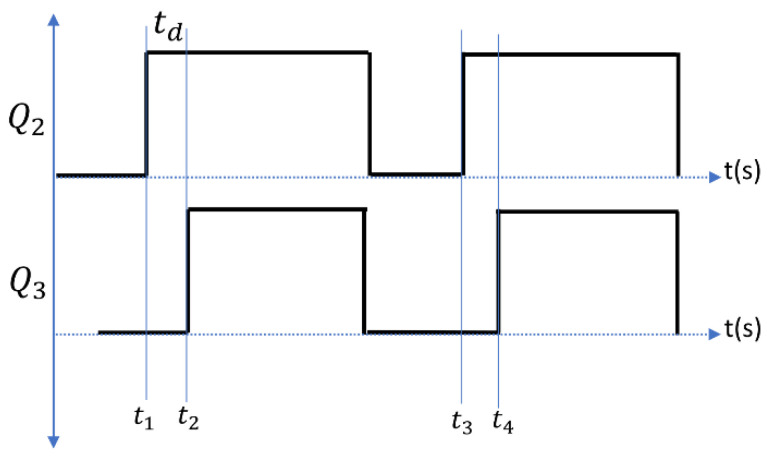
*Q*_2_ and *Q*_3_ MOSFETS ON/OFF condition.

The modelling of the EDM process as shown in Figure 6 was simulated in MATLAB 2021a—a Simulink environment with the process parameters as listed in Table 1. The inductor and capacitor values of the buck converter were fixed as per the calculations discussed in Section 2 for the simulated parameters. The gap voltage and current for the low- and high-spark frequency are already shown in Figure 3. The MOSFET switch $Q_{1}$ was pulsed with a duty cycle of 64% to give the $V_{in}$ as 160 V. The simulated parameters were chosen based on the experimental data conducted in [26,27]. The MOSFETs $Q_{2}$ and $Q_{3}$ were switched on with a delay $t_{d}$ period shift, with both having the same pulse spark frequency as shown in Figure 7. This time shift ensured all three phases in the EDM process. Since the Equations (12) and (13) were valid under certain conditions, the delay time $t_{d}$ was fixed as 2 µs for all the sampling frequencies and the discharging time was chosen as being up to 450 µs.

### 4.2. EDM Process and Model Outputs

The cycle of the EDM process was performed in terms of the three phases of ignition, discharge, and recovery in a sequential manner. The input voltage and gap voltage were selected with a small difference. The proposed model was evaluated with an assumption of a noise-free environment. The gap voltage and current for the various spark frequency values were obtained during the process exactly to represent the EDM process phases. With the help of the experimental data given in [27], the model was verified. All the processes were carried out with steel work pieces and copper electrodes with their material property a constant α in Equation (12). The actual values fetched from reference [27] were modelled using the proposed model and compared with the actual values to analyze the accuracy of the modelling, as shown in Table 2.

**Figure 8:**
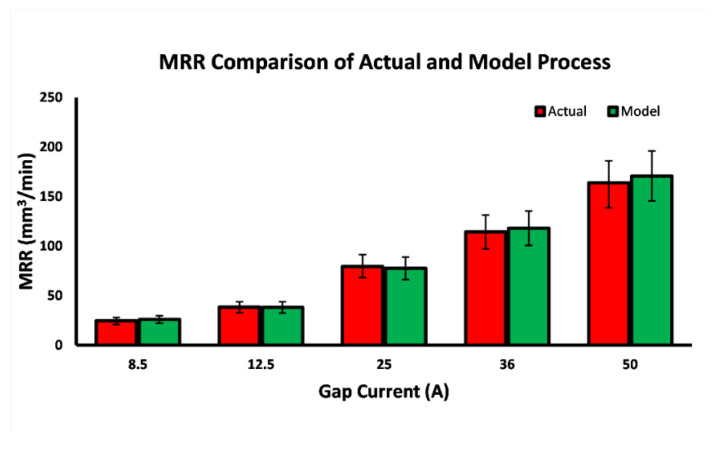
Material removal rate (*MRR*) comparison of the actual process and simulated modelling.

The comparison between the actual and model outputs is shown in Table 2. There were about 50 processes with varying discharge times for the different gap currents manually carried out. The material removal rate obtained using the simulated model for the different gap currents were varied with the actual process outputs in an acceptable error value. The minimum error of 0.84% for the gap current value 12.5 A and the maximum error of 4.2% for the gap current value of 50 A were observed as shown in Figure 8. This average error was calculated through a number of processes conducted for different discharge times and spark frequencies. Almost the maximum material removal rate was obtained in the range of the discharge time from 50 μs to 150 μs, with a spark frequency range of 125 kHz to 2.21 kHz as given in Table 2. It was assessed that the model behaved as an actual process with a maximum error of 4.2%.

Table 2 presents only the manual searching of the optimal value of the discharge time for which the maximum material removal rate was obtained. If any one of the process parameters of the EDM process changed, then the optimal point for the maximum *MRR* would be changed. For a new optimal point, the manual searching should then be conducted again. This optimal point was dynamic under the dynamic conditions of the EDM process, but there was no assurance that the current optimal value was a true optimal value for that particular process under dynamic conditions. To alleviate the aforementioned problem, an online extremum-seeking control algorithm—as already discussed—can be used to find the optimal discharge time online under dynamic conditions.

### 4.3. Extremum-Seeking for Optimal Discharge Time

The high pass filter and demodulation section together can be used to extract the gradient information as discussed in the previous section. The input can be estimated using a controller with a gain K after filtering out the higher order harmonics using a low pass filter. The amplitude a = 0.5 and the controller gain K = 100 were fixed for having a nominal convergence speed and a nominal final error deviation around the optimal point in a trial-and-error method using Equation (19). Owing to a larger phase change at higher frequencies by the EDM process, the perturbation frequency ω was chosen as a low value to avoid unstable behavior of the closed-loop system [34].

**Figure 9:**
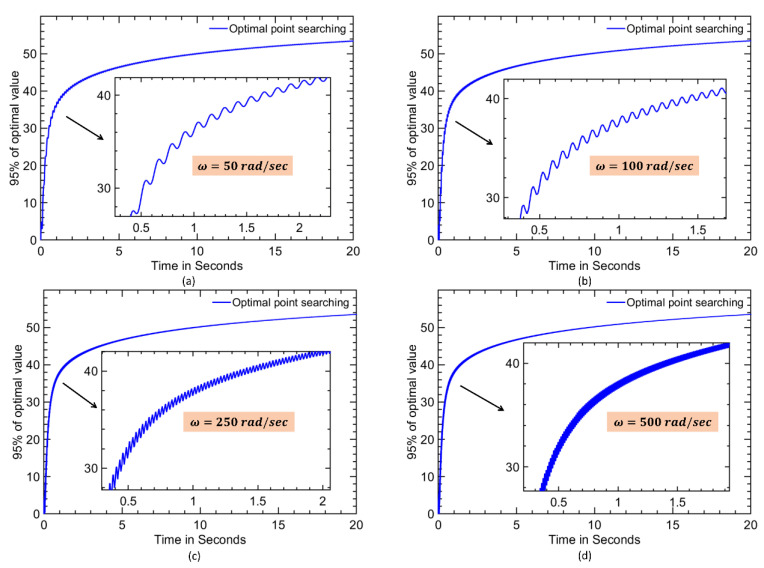
Optimal perturb frequency ω searching for fast settling with a gap current of 8.5 A and (**a**) *ω* = 50 rad/s, (**b**) *ω* = 100 rad/s, (**c**) *ω* = 250 rad/s, and (**d**) *ω* = 500 rad/s.

**Figure 10:**
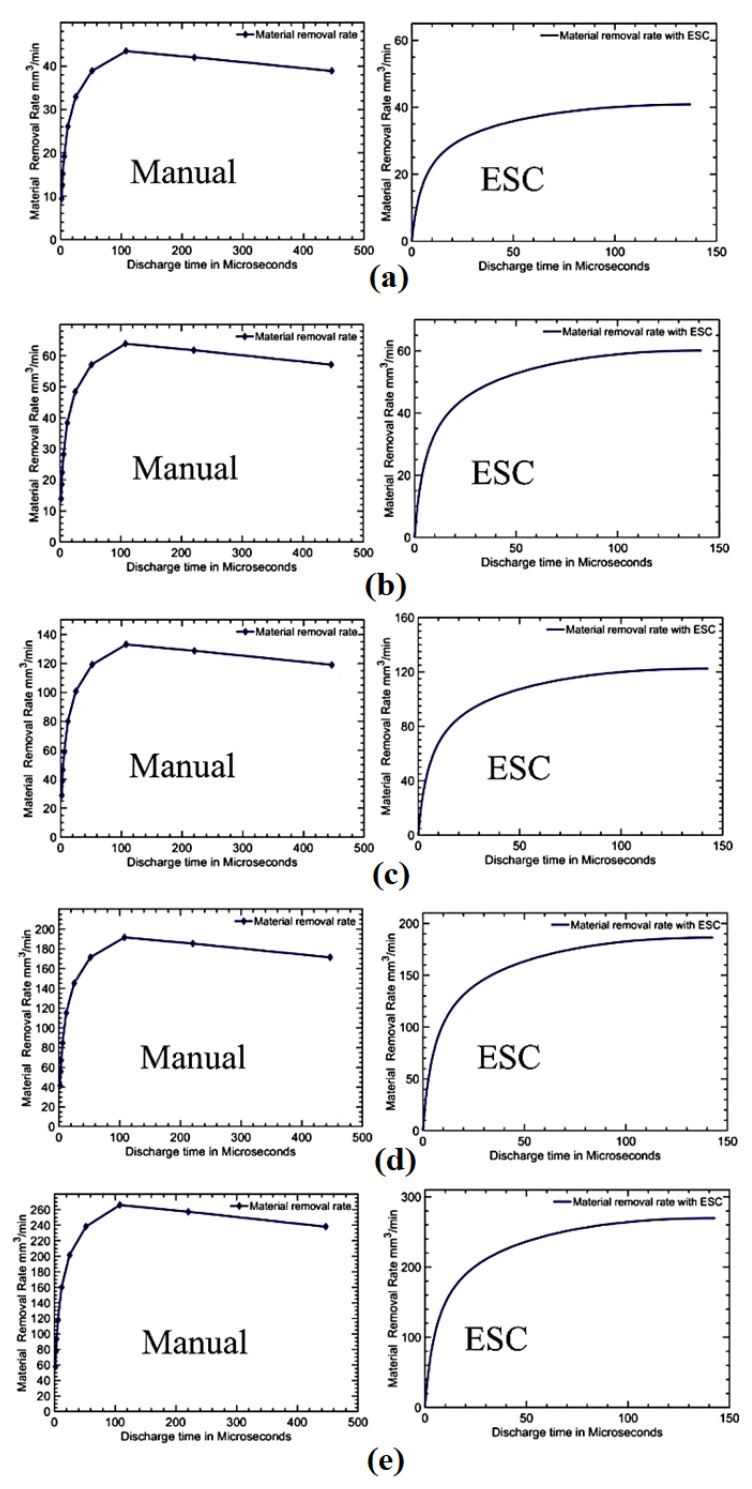
Comparison of discrete manual searching (**left**) and extremum-seeking optimal searching (**right**) for gap currents (**a**) 8.5 A, (**b**) 12.5 A, (**c**) 25 A, (**d**) 36 A, and (**e**) 50 A.

**Figure 11:**
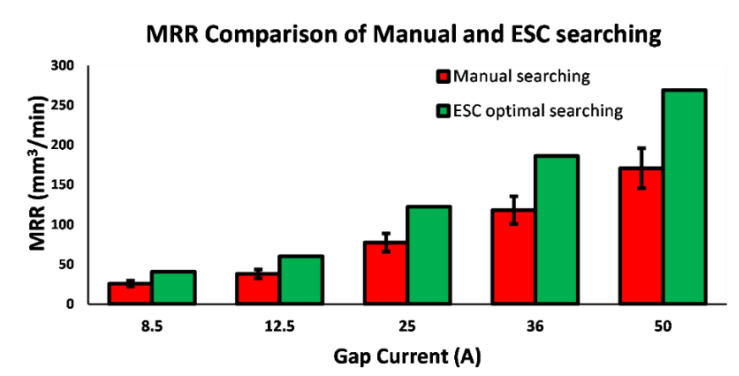
*MRR* comparison of manual and ESC searching.

Since the convergence speed depends on frequencies [34], the optimum convergence speed can be found by plotting responses to 95% of its final maximum removal with a varying perturbation frequency of *ω*. The frequency at which the output reaches the first maximum is considered to be an optimum value. Since the terms a and K were involved in the 5% error in their final value, 95% of the final value was chosen for a fast settlement measurement. It was assumed that all the searching processes began from a zero $t_{on}$ as the worst-case scenario. The better convergence speed was obtained at a frequency *ω* = 200 rad/s with the least settling time of 20.46 s and seeking a 95% final value for the different perturbing frequencies of 50, 100, 200 and 500 rad/s with a gap current of 8.5 A, as shown in Figure 9. The arrow in the Figure indicates the magnified form of the response curve. The discrete manual searching and extremum-seeking optimal searching for the various gap currents, such as 8.5 A, 12.5 A, 25 A, 36 A and 50 A, were computed as shown in Figure 10. The optimal on time value with a maximum *MRR* was computed based on an ESC control and were computed as shown in Figure 11 and Table 3.

**Figure 12:**
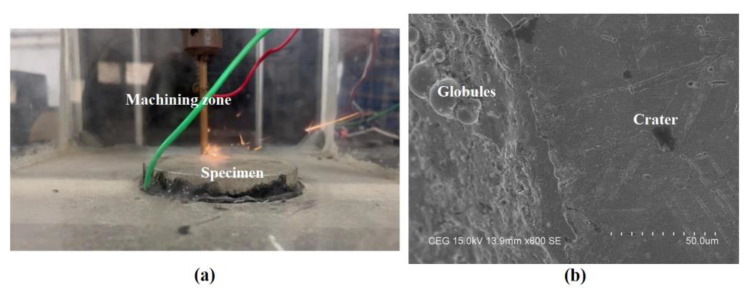
(**a**) EDM arrangement and (**b**) machine surface using SEM.

After fixing the *ω*, the optimal search was carried out using a perturb and observe manner in the ESC loop for the different spark frequencies. Once the maximum was reached, the searching was stopped, and the final maximum material removal rate was settled with an optimum value of the discharge time. The comparison of the discrete manual optimal searching and extremum-seeking optimal searching is shown in Figure 10 for the different gap current values. The optimal discharge time and its corresponding maximum material removal rate are tabulated in Table 3 with a discharge time of 2 μs to 446.5 μs and a spark frequency of 125 kHz to 2.21 kHz. Table 3 shows the optimal on time with the corresponding *MRR* that were obtained from the ESC modelling. The experimental trials were also performed to further analyze the effectiveness of the ESC approach. The performance of the proposed extremum-seeking optimal searching was compared with the manual searching and is plotted in Figure 11. When compared to the average number of manual trial operations, the extremum-seeking optimal searching delivered a 57% higher rate of material removal, but compared to the most effective manual searching method, it was 1.2% more efficient. The developed ESC approach was implemented in the EDM arrangement shown in Figure 12. The tool electrode and specimen were separated by an air gap and immersed in a dielectric medium [35]. The discharge sparks were formulated in the machining zone of the EDM arrangement. The tool was connected with a negative polarity whereas the specimen was connected with a positive polarity [36]. The craters were created over the machined surface by a developed thermal spark energy [37]. These craters were observed along with resolidified globules over the machined surface in the EDM process. All the trials were conducted three times and the average of those values were fixed as a final value under the chosen gap current of 8.5 A, 12.5 A, 25 A, 36 A and 50 A. The AISI 304 stainless steel specimens were machined with a copper tool electrode in the presence of EDM oil as the dielectric medium under a flushing pressure of 2 bar. The *MRR* was calculated by finding the weight difference of the specimens before and after the machining process. It was observed that the experimental values were similar to the simulation results but with less errors. The craters produced by the EDM process were obtained by a scanning electron microscope (SEM: JEOL JAPAN, Jeol-6490 JED-2300) as shown in Figure 12.

## 5. Conclusions

In this paper, a low-power EDM process model was designed and developed. It was successfully simulated with assigned process parameters and verified with actual EDM process experimental data. The possibility of an extremum-seeking control on the *MRR* for optimal input searching was studied and implemented successfully. The following conclusions were made:

➢ The simulated model gives less errors from 0.82% to 4.2% with the actual process output.
➢ The possibility of extremum-seeking control in the search for an online optimal point with less error and more convergence speed was studied.
➢ An extremum-seeking optimal search offers a 57% greater material removal rate against the average number of manual trial processes, but it offers 1.2% more efficiency than the best manual searching process.
➢ The experimental validation also proved that the ESC can produce large *MRR* by tracking the extremum control.
➢ The present study was limited to only *MRR*, but it is also possible to implement such algorithms for more than one response parameter optimization in future studies. In such cases, the performance measures of the process can be further enhanced which can be used for real-time complex die- and mold-making processes using EDM.

## Figures and Tables

**Table 1:** Process parameters.

Parameters	Value
Supply Voltage and Frequency	240 V and 50 Hz
Filter Capacitor	1 F
Ignition Resistor and Inductor	2 Ω and 0.1 mH
Switching Frequency for Buck converter	30 KHz

**Table 2:** EDM process and model outputs.

Process	*I_g_* (A)	*F_s_* (kHz)	*T_on_* (µsec)	*MRR* (mm^3^/Min)	
				Actual	Model
1	8.5	125	2.0	8	8.77
2	8.5	111.1	3.0	11	11.74
3	8.5	100	4.0	16	14.14
4	8.5	83.33	6.0	21	17.81
5	8.5	55.55	12.0	23	24.27
6	8.5	32.25	25.0	31	30.57
7	8.5	17.24	52.0	36	36.12
8	8.5	8.77	108.0	38	40.35
9	8.5	4.41	220.8	33	39.03
10	8.5	2.21	446.5	29	36.10
11	12.5	125	2.0	12	12.90
12	12.5	111.1	3.0	16	17.27
13	12.5	100	4.0	20	20.80
14	12.5	83.33	6.0	31	26.20
15	12.5	55.55	12.0	43	35.69
16	12.5	32.25	25.0	48	44.95
17	12.5	17.24	52.0	52	53.12
18	12.5	8.77	108.0	54	59.34
19	12.5	4.41	220.8	54	57.40
20	12.5	2.21	446.5	54	53.09
21	25	125	2.0	26	26.26
22	25	111.1	3.0	31	35.16
23	25	100	4.0	46	42.34
24	25	83.33	6.0	60	53.34
25	25	55.55	12.0	81	72.66
26	25	32.25	25.0	99	91.51
27	25	17.24	52.0	126	108.15
28	25	8.77	108.0	126	120.81
29	25	4.41	220.8	110	116.86
30	25	2.21	446.5	90	108.08
31	36	125	2.0	39	39.98
32	36	111.1	3.0	53	53.53
33	36	100	4.0	64	64.47
34	36	83.33	6.0	72	81.20
35	36	55.55	12.0	111	110.62
36	36	32.25	25.0	137	139.32
37	36	17.24	52.0	161	164.65
38	36	8.77	108.0	181	183.94
39	36	4.41	220.8	175	177.92
40	36	2.21	446.5	151	164.55
41	50	125	2.0	57	57.84
42	50	111.1	3.0	77	77.44
43	50	100	4.0	82	93.27
44	50	83.33	6.0	143	117.48
45	50	55.55	12.0	170	160.04
46	50	32.25	25.0	218	201.57
47	50	17.24	52.0	250	238.21
48	50	8.77	108.0	221	266.11
49	50	4.41	220.8	221	257.41
50	50	2.21	446.5	200	238.07

**Table 3:** Extremum-seeking of the *MRR* for gap currents.

Process	*I_g_* (A)	ESC			
		Optimal on Time (µs)	Maximum *MRR* (mm^3^/min)	Experimental *MRR* (mm^3^/min)	Error (%)
1	8.5	136	40.86	37.85	7.37
2	12.5	140	60.11	55.68	7.35
3	25	141	122.4	112.65	7.97
4	36	142	186.3	174.92	6.11
5	50	142	269	249.5	7.25

## Data Availability

Not applicable.
